# Towards patient-centred cancer care: cross-cultural validity and responsiveness of the Turkish Integrated Palliative care Outcome Scale

**DOI:** 10.1186/s12955-020-01535-5

**Published:** 2020-09-22

**Authors:** Mevhibe B. Hocaoglu, Nilay Hepgul, India Tunnard, Emine Meltem, Hande Efe, Buse Ataoglu, Yeliz Lerzan Baybar, Merve Kınacıgil, Husam Elsharairi, Irene J. Higginson

**Affiliations:** 1 Cicely Saunders Institute of Palliative Care, Policy & Rehabilitation, Florence Nightingale Faculty of Nursing, Midwifery & Palliative Care, King’s College London, London, UK; 2 Faculty of Medicine, Eastern Mediterranean University, Famagusta, North Cyprus Cyprus

**Keywords:** Patient reported outcome measures, Palliative care, Psychometrics, Validation study, Cancer

## Abstract

**Background:**

A valid measure to describe the most important needs and concerns of people with life-threatening illnesses is missing in Cyprus. Our aim was to adapt and test the cross-cultural validity and responsiveness of the Integrated Palliative care Outcome Scale (IPOS) in a cohort of Turkish speaking cancer patients.

**Methods:**

The IPOS (English) patient-reported measure was translated into Turkish following published guidelines including, 2 independent forward, 2 independent blind backward translations, expert panel review by 7 members and field testing with 11 cognitive interviews (5 patients and 6 specialists) and final approval of the copyright holder. Consecutive cancer patients (*n* = 234) seen by the community palliative care services were recruited from Help Those with Cancer Society (KHYD); of those 82 were followed-up. The instrument was administered by personal interview. Confirmatory Factor Analysis was used to validate the factor structure of Turkish IPOS. Internal consistency reliability of the subscales was evaluated by Cronbach’s alpha and Intraclass Correlation Coefficient respectively. Validity was assessed by calculating Pearson’s correlation coefficient (*r*) between Turkish IPOS scores and Turkish version of EQ-5D-3L - a validated generic measure of health status developed by the EuroQol Group.

**Results:**

Turkish IPOS is conceptually and semantically equivalent to the English version and linguistically valid. The CFA was inconclusive for the three factor structure due to low sample size, as the SRMR and CFI tests only approached the defined minimums warranting further investigation. There were low levels of missing values, and no c*eiling or floor effects.* The Physical (α = 0.91) and the Social and Quality of Care Issues (α = 0.75) sub-scales showed good internal consistencies, however Emotional sub-scale showed poor internal consistency (α = 0.64). The reliability of the Physical (ICC = 0.51, 0.45–0.56 95% CI) and Social Quality of Care Issues (ICC = 0.50, 0.42–0.57 95% CI) were moderate. Poor internal consistency (α =0.64) and reliability (ICC = 0.31, 0.24–0.39, 95% CI) was obtained for Emotional Subscale. Construct validity was evidenced through significant correlations in the predicted directions and strength with EQ-5D. Turkish IPOS showed higher needs and concerns in participants at more advanced stages than those at earlier stages of cancer. The standardized response mean (SRM) of − 0.94 suggested large internal responsiveness to clinical change.

**Conclusion:**

Turkish IPOS is a clear, relevant, acceptable measure and responsive to the needs and concerns of cancer patients, observing regional differences, it may have implications for use in other Turkish speaking communities. Future studies are needed to clarify the factor structure, assess its external responsiveness and to improve the properties of its Emotional subscale.

## Background

A comparative review of palliative care development in the Middle Eastern Cancer Consortium (MECC) member countries identified ‘*lack of awareness and understanding of palliative care needs at public, government and professional levels*’ as barriers to provision of palliative care to cancer patients in this region [1]. These observations maintain their relevance today for cancer patients living in MECC member countries including the setting for this study, Cyprus. Palliative care as ‘ *… an approach that improves the quality of life of patients and their families … through the prevention and relief of suffering by means of early identification and impeccable assessment and treatment of pain and other problems, physical, psychosocial and spiritual*’ [2] is rarely recognized [3]. As a concept, palliative care is only equated with management of pain in cancer and available only in oncology inpatient and intensive care units [4].

Within Cyprus, resources for health services including community home-based services are scarce and receive no or little government support. The health expenditure is the lowest compared to other European Union member countries and 25% of the population has no access to public health services [5]. Early palliative care as cost-effective intervention in cancer patients has already been demonstrated [6]. Evidence of the effectiveness and cost-effectiveness of palliative care services may promote their integration for persons living with all serious illnesses in Cyprus. After years of lobbying, local cancer charities have been formally authorized to deliver community home-based palliative care services in Cyprus [4, 7]. This recognition may serve as a ‘*catalytic action*’ [8] that may increase the coverage of the palliative care services beyond inpatient and intensive care unit settings to the community. The charities must support home services through fundraising and ensure their resources address the most important concerns early. This has necessitated availability of a brief and valid patient-reported outcome measure (PROM) to describe the most important concerns of people with living with cancer.

Routine use of PROMs can improve the quality and relevance of services [9] and also can help build awareness in healthcare providers about the most important needs of patients and families [10]. The Integrated Palliative care Outcome Scale (IPOS) is a valid brief PROM that addresses the most important concerns such as symptoms, information needs, practical concerns, anxiety and low mood, family anxieties and overall feelings of being at peace of persons living with serious illnesses, such as cancer [11, 12]. IPOS has been used with cancer patients [13, 14] and adapted to many cultures [15–17].

In this study, we first translated and culturally adapted IPOS into Turkish, and evaluated the cross-cultural validity and responsiveness of IPOS in Turkish speaking community of cancer patients in Cyprus. The findings of the study could have implications for millions of patients living with serious illnesses around the world who have Turkish as their native language [18].

## Methods

The study commenced after attaining permissions from the POS Development Team as the copyright holder of IPOS, the EuroQol Group for use of Turkish version of EQ-5D-3L. Ethical approval was obtained from the Eastern Mediterranean University Publication and Research Ethics Committee (ETK00–2017-103). Written informed consent was obtained from all participants included in the study.

### Participants and procedures

Patients seen by the community palliative care team including new or old referrals, of Help Those with Cancer Society in Cyprus (KHYD), aged 18 and above were eligible to participate in the study. Patients who were not referred to the community palliative care services, or were younger than 18, were excluded from the study. Invitation and recruitment was consecutive and stopped once the target size of at least 10 cases per item [19] were reached. Two hundred and thirty-four consenting patients were asked to complete the Turkish IPOS alongside the EQ-5D-3L. For follow-up, 82 participants were asked to complete the Turkish IPOS a second time during their next routine visit. Due to unavailability of resources, the follow-up visit and assessment could only carried out in the next scheduled routine visit by the community palliative care team rather a specified time period. Data were collected by the 4 community nurses during routine home visits.

Patients were also asked to complete questions about their age, gender, education, marital status, occupation, number of children, how they were meeting treatment and care expenditures, their sources of support and co-morbidities. Clinical information on the primary tumour site including stage was extracted by the Community Palliative Care Team (CPCT) from their membership registration files. The testing and reporting of the measurement properties of the Turkish IPOS followed the Consensus-based Standards for the selection of health Measurement Instruments (COSMIN) recommendations [20].

### Measures/questionnaires

#### Integrated Palliative care Outcome Scale (IPOS)

IPOS is a 10 question, 17-item brief PROM addressing symptoms, information needs, practical concerns, anxiety, low mood, family anxieties and overall feeling of being at peace of persons living with life-threatening illnesses. It is scored on a 5-point Likert scale (0–4) with higher scores indicating an overwhelming presence of symptoms and needs not addressed. Patients may also list their main problems and concerns and any additional symptoms. The 7 day patient version recommended for use in community-based services was used in this study [21]. An earlier study identified Physical Symptoms, Emotional Issues and Support (Social issues and Quality of Care) as three sub-scales of IPOS [11].

#### EuroQol Group’s Generic Health Status Preference based Measure - EQ-5D-3L

*EQ-5D-3L* evaluates a person’s health status based on 5 dimensions: mobility; self-care; usual activities; pain/discomfort; and anxiety/depression. It is scored on a scale from 1 to 3 where 1 represents ‘no problems’, 2 denotes ‘some problems’, and 3 ‘extreme problems.’ It is accompanied by a Visual Analogue Scale (EQ VAS) which records the patient’s self-rated health on a vertical VAS with the endpoints ‘Best imaginable health state’ and ‘Worst imaginable health state’. Turkish version of EQ-5D-3L has been validated in cancer patients [22] and therefore was chosen as comparative measure.

### Translation and cross-cultural adaptation

The ‘*Manual for cross-cultural adaptation and psychometric validation of the IPOS development group*’ [23] was rigorously followed for the translation and cross-cultural adaptation. Literature review and consultations with patient, their families and clinicians was used to establishing IPOS’ face and content validity. Face and content validity were reconfirmed in the validation phase of the study by the free text entries of respondents.

Four translators and two mediators followed the independent forward- blind backward translation method to translate IPOS (English) to Turkish. Two translators who carried out the two independent forward translations, were native in Turkish and fluent in English. One of the translators were an English teacher, the second one was a general practitioner. Two translators, who carried out the blinded backward translations were native English speakers, who have moved to Cyprus from the UK, and were fluent in Turkish. Both were English teachers. The mediators were two cancer patients. The expert review panel included all four translators and two mediators, and the first author. The expert review panel reviewed and synthesized the translations, and prepared the pre-final Turkish IPOS for piloting (cognitive testing).

A total of 5 cancer patients and 6 clinicians including nursing staff and counsellors, completed the pre-final Turkish IPOS in order to test the alternative wording and to check understandability, interpretation, and cultural relevance of the translation [24]. Probes such as ‘What does the term *practical problems* mean to you?’, ‘In your own words can you repeat what this question is asking?’, ‘Was that question easy or hard to answer?’ and ‘I can see that you have chosen *moderately* or 2 as your response or score, why is that?‘were used [24]. Participants found the questionnaire easy to understand. Completed reports on the general information on participants, translators and expert review panel members, summary of all phases of the process and on the synthesis of the two forward translations and the two backward translations together was collated in a final report. These documents with the Turkish IPOS Pre-Approval Version as agreed by the research team and the copyright holder, were sent to POS Development Team at King’s College London for review and final approval before proceeding to validation phase.

### Statistical analysis

Descriptive and multivariate analyses were carried out to present participant characteristics and to assess data completeness, distribution, internal consistency, reliability, validity and internal responsiveness of Turkish IPOS using IBM SPSS Statistics 25.0 and Amos 25.0 [25]. Pairwise deletion was used in multivariate statistical analyses [26]. Participant characteristics were summarized as percentages of the total participants. Mean or median with standard deviation or range of values were presented where relevant. Data completeness was assessed by Missing Value Analysis (MVA). For acceptability, means, standard deviation, range of values, skewness, kurtosis as well as the ceiling and floor effects were assessed for the total scores as well as for the three sub-scales of IPOS. Content validity were re-confirmed through patient responses to two free-text parts of the Turkish IPOS, one about main problems and concerns, and the second on any other symptoms not listed.

A first order Confirmatory Factor Analysis (CFA) was used to assess how well the hypothesized 3-factor model fits the observed data. The model parameters were estimated using Full Information Maximum Likelihood (FIML) method to make maximal use of all data [26]. Post-hoc modifications were undertaken to improve model fit. As the chi-square statistic has been shown to fail to discriminate between good fitting models and poor fitting models in small sample sizes (*n* ≤ 250) [27], combinational rules and cut-off criterion for fit statistics that minimize Type I and Type II errors in small samples were considered [28]. Comparative Fit Index (CFI) and Standardized Root Mean Square Residual (SRMR) were used to evaluate the model fit with CFI ≥ 0.95 and SRMR< 0.09 suggested good fit.

Internal consistency of each sub-scale was evaluated with Cronbach’s α coefficient [29] where α values in the range of 0.70–0.90 is indicative of good internal consistency [30]. In order to estimate the reliability, Interclass Correlation Coefficients (ICCs) using two-way mixed effect model was obtained and ICCs for single measurement are reported. The choice of the model was informed by the fact that multiple administrators from a selected pool carried out the assessments and consistency of measurement was critical [31]. ICCs less than 0.5 are indicate poor, between 0.5 and 0.75 indicate moderate, 0.75 and 0.9 are indicate of good, and above 0.90 indicate excellent reliability [31].

Construct validity where conceptual convergence and divergence between Turkish IPOS and EQ-5D-3L items were evaluated with bivariate correlation analysis, where Pearson’s correlation coefficient (*r*) between 0 and 0.2 indicated weak, 0.2–0.4 low, 0.4–0.6 moderate and 0.6–0.8 strong relationship given statistical significance. Known groups (discriminant) validity was assessed by independent samples t-test comparing total and sub-scale scores in participants with early (Stages I-II) versus advanced cancer (Stages III-IV).

Standardized Response Mean (SRM) was calculated to estimate change in scores standardized relative to variability between the participants to assess internal responsiveness [32]. Baseline and follow-up scores were compared using paired t-test. Bonferroni corrected *p* ≤ 0.01 was used for significance to account for multiple comparisons.

## Results

Literature and consultations confirmed that IPOS covered all of the relevant concepts in relation needs and concerns and had face and content validity. Pain and emotional difficulties emerged as the most prevalent issues. In the 2 forward translations there were differences in the grammatical tense used; translators used present continuous tense, but the mediators used past tense. The item on practical problems resulting from illness was particularly challenging, therefore the word ‘practical’ was removed and the question revised to ask about ‘personal and financial problems’ caused by illness. The 2 blind backward translations produced from final reconciled forward translation of Turkish IPOS were both similar with no significant differences in meaning. The expert panel reviewed all versions to improve clarity and produced the pre-final Turkish IPOS.

Following cognitive testing, instructions were reworded and further shortened. The response options were generally clear and well understood; the only difficulty was with ‘occasionally’ which was suggested to be changed to ‘zaman zaman’ rather than ‘ara sıra’. Patients struggled with the information item which was revised to emphasize that the question was not about the knowledge the patients possessed but the information given to them. Turkish IPOS Pre-approval Version was sent to the Cicely Saunders Institute POS Team for independent review. Following this and minor amendments, Turkish IPOS was produced and validation study was initiated.

For the validation study, data collection took place between July 2017 and October 2018. Participants were mostly women (73.5%), almost all were struggling with treatment expenses (91.5%) and most received support from family members (83.3%). Of the 43.6% who reported having comorbidities, 50% had hypertension and 28.4% had diabetes (Table 1). Fifty three percent of the participants had advanced cancer and breast cancer was the most prevalent (48.3%).

**Table 1:** Participant clinical and socio-demographic characteristics (*n* = 234)

Characteristics	Measure	Value
*Age*	Mean (SD)	58.23 (12.45)
	Median (min, max)	60 (19.86)
*Sex*	Woman%	73.5
*Highest level of Education*		
University and above	%	13.7
Highschool	%	30.8
Primary school	%	26.9
No schooling	%	1.7
*Marital status*	Married/Have a partner	73.1
	Single or Divorced %	26.5
*Have children*	Yes%	87.6
*Payment for Health*		
Social insurance	Yes%	64.5
Private Health Insurance	Yes%	15.4
*Experiencing difficulties in meeting health expenses*	Yes%	91.5
*Source of Support*		
Family members play a role in supporting the patient	Yes%	83.3
Friends play a role in supporting the patient	Yes%	43.2
Professionals support the patient	Yes%	1.3
Civil Society Organizations support the patient	Yes%	82.1
No need for support	Yes%	1.7
No support	Yes%	1.7
*Source of Comfort*		
Feeling Better / Family	Yes%	81.2
Feeling Better / Friends	Yes%	53.4
Feeling Better / Social Media	Yes%	14.5
Feeling Better / Being alone	Yes%	7.3
*Comorbidities*	Yes%	43.6
Hypertension	%	50
Diabetes	%	28.4
Thyroid	%	6
Asthma	%	2
Other conditions:	%	13.6
*Disease stage (MV)*		
I (relatively small and contained within the organ it started in)	%	6.8
II (bigger than I but cancer has not started to spread into the surrounding tissues only to lymph nodes)	%	30.3
III (started to spread into surrounding tissues and there are cancer cells in the lymph nodes in the area)	%	34.2
IV (metastatic - has spread from where it started to another body organ)	%	18.8
*Anatomical Site of Origin of Cancer (Top 7)*		
Breast	%	48.3
Lymph Nodes	%	6.8
Colon	%	6.8
Prostate Gland	%	6
Bronchus and Lung	%	3.8
Thyroid	%	3.8
Uterus	%	3

**Fig. 1:**
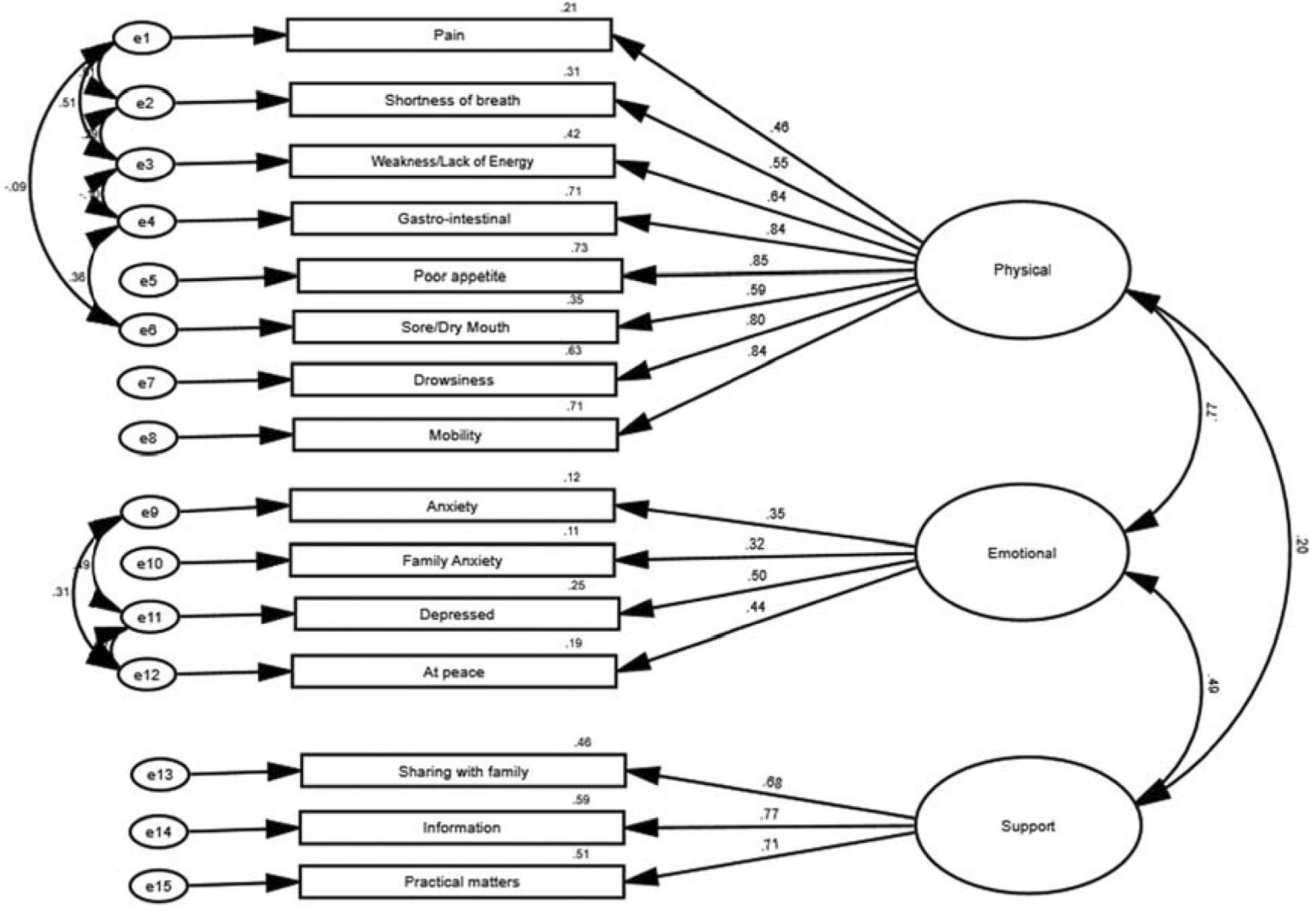
Cross-cultural validity: Standardized measurement model following post-hoc modification for Confirmatory Factor Analysis (*n* = 234)

The IPOS total scores were normally distributed (Fig. 1). There were no ceiling or floor effects for the total score or for any of the sub-scale scores (Table 3)

MVA identified four items with higher number of missing responses in baseline assessment (9.4–7.7%) (Table 2). As the data were not missing completely at random (Little’s MCAR test χ^2^ = 291.512, DF = 184, *p* < 0.0001), listwise deletion or imputation of missing values was not undertaken. Patients at moderate stages of their illness (Stage II and III) had more than 5% of responses missing for these items. Only two items were missing 1 response each in the follow-up assessment.

**Table 2:** Data Completeness: Numbers and percentages of complete responses and non-responses in baseline and follow-up assessments

*Item*	Baseline Assessment		Follow-up Assessment	
	*Number of complete responses*	*Number of non-response (%)*	*Number of complete responses*	*Number of non-response (%)*
Depression	212	22 (9.4)	82	0 (0.0)
Information	213	21 (9)	82	0 (0.0)
Anxiety of Friends and Family	215	19 (8.1)	82	0 (0.0)
Sharing Feelings with Family and Friends	216	18 (7.7)	82	0 (0.0)
Constipation	230	4 (1.7)	81	1 (1.2)
Sore or Dry Mouth	230	4 (1.7)	82	0 (0.0)
Feeling at Peace	230	4 (1.7)	82	0 (0.0)
Drowsiness	231	3 (1.3)	82	0 (0.0)
Shortness of Breath	231	3 (1.3)	82	0 (0.0)
Weakness or Lack of Energy	231	3 (1.3)	82	0 (0.0)
Pain	232	2 (0.9)	82	0 (0.0)
Nausea	232	2 (0.9)	82	0 (0.0)
Vomiting	232	2 (0.9)	81	1 (1.2)
Poor Mobility	233	1 (0.4)	82	0 (0.0)
Poor Appetite	233	1 (0.4)	82	0 (0.0)
Anxiety about Illness or Treatment	234	0 (0.0)	82	0 (0.0)
Practical Problems	234	0 (0.0)	82	0 (0.0)

**Fig. 2:**
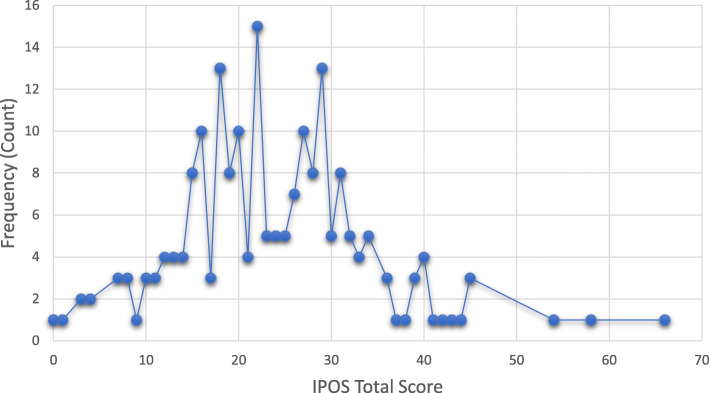
Distribution of Turkish IPOS total scores (*n* = 204) where 0 = No needs, concerns and/or symptoms, 68 = Highest needs, concerns and/symptoms

CFA fit indices were CFI = 0.756 and SRMR = 0.0002 indicated poor fit of the model to the data (χ^2^ = 496.369, df = 87, χ^2^/df = 5.705, *p* < 0.0001) and post-hoc modifications were made. Post-hoc modification involved adding in covariances of error terms based on theoretical and relevant associations of items measured. The model fit improved where CFI = 0.855 and SMRM = 0.0002 (χ^2^ = 321.874, df = 78, χ^2^/df = 4.127, *p* < 0.0001). Even though the CFI and SMRM parameters approached the minimums, they were not within the required defined parameters recommended for small samples. For this reason, the CFA was inconclusive and cross-cultural validity could not be confirmed or negated. The standardized parameter estimates of the modified model are shown in Fig. 1. Physical factor accounted for 73–21% of variance, Emotional factor for 25–11% and Social Impact and Quality of Care (Support) for 50–32% of the relevant IPOS items. Emotional subscale, had poorly loading items where Anxiety and Anxiety of Friend and Family had factor loadings of 0.32 and 0.35 respectively (see Table 3 and Fig. 2 for distribution of scores).

**Table 3:** Acceptability: Distribution of Turkish IPOS scores

Domain (n)(Possible Min-Max Score)	MeanAge(SD)	Mean	SD	Range	Skewness	Kurtosis	CeilingEffect % (n)	Floor Effect % (n)
IPOS Total Score (*n* = 204)(0–68)	58.20 (12.59)	23.70	10.09	0–64	0.57	1.31	0.50 (1)	0.50 (1)
Physical Sub-scale (*n* = 226) (0–32)	58.23 (12.43)	8.92	5.91	0–32	0.85	1.06	0.40 (1)	2.70 (6)
Emotional Sub-scale (*n* = 211)(0–16)	58.32 (12.59)	7.11	2.81	0–16	0.36	0.62	0.50 (1)	0.90 (2)
Social and Quality of Care Sub-scale (*n* = 210)(0–12)	58.26 (12.66)	5.56	2.66	0–12	−0.32	−0.57	0.50 (1)	0.90 (2)

The Emotional sub-scale showed poor internal consistency (α = 0.64), three of the items were worthy of retention but deletion the item on anxiety of family and friends would increase α to 0.75. The reliability of this subscale was also poor (ICC = 0.31, 0.24–0.39, 95% CI). The Physical subscale showed good internal consistency (α = 0.91) but moderate reliability (ICC = 0.51, 0.45–0.56 95% CI). Social and Quality of Care Issues subscale showed good internal consistency (α = 0.75) and marginally moderate reliability (ICC = 0.50, 0.42–0.57 95% CI).

The participants listed feeling worried, financial difficulties, lack of support, impact of cancer on the family on activities of everyday life as their main concerns in response to the open-ended first question in IPOS. As these topics were all covered by IPOS, its content validity was reconfirmed.

Correlations with EQ-5D-3L domains were significant and, mostly in the directions and strength predicted (Table 4). For example, IPOS Emotional subscale, had low correlations with EQ-5D Mobility, Self-care, Usual Activity, slightly higher but still low correlations with Pain/Discomfort and Anxiety/Depression and moderate negative correlation with self-rated health.

**Table 4:** Construct Validity: Bivariate Correlational Analysis (Pearson’s rho) of IPOS and EQ-5D

		Turkish IPOS Domains			
		IPOS Total Score	Physical Subscale	Emotional Subscale	Social Impact & Quality of Care Subscale
**EQ-5D-3L**	Mobility	0.237^a^	0.197^a^	0.224^a^	0.236^a^
	Self-care	0.461^a^	0.336^a^	0.258^a^	0.443^a^
	Usual activity	0.467^a^	0.417^a^	0.261^a^	0.332^aa^
	Pain/Discomfort	0.521^a^	0.418^a^	0.354^aa^	0.369^a^
	Anxiety/Depression	0.465^a^	0.311^a^	0.363^a^	0.501^a^
	Self-rated Health (VAS)	−0.459^a^	−0.335^a^	−0.429^a^	−0.334^a^

^a^Correlation is significant at the 0.01 level (2-tailed)

Known-group validity was indicated as there was a significant difference in the total scores for early (M = 22.03, SD = 10.39) and advanced (M = 25.51, SD = 9.27) stages; t (128.526) = − 2.274, *p* = 0.025. Similarly, participants at earlier disease stage had significantly lower Social and Quality of Care sub-scores (M = 4.87, SD = 2.75) than participants at advanced stages of cancer (M = 6.41, SD = 2.25), t (123.635) = − 3.955, *p* < 0.0001. The Physical and Emotional sub-scale scores for participants at early and advanced stages were not significantly different.

**Fig. 3:**
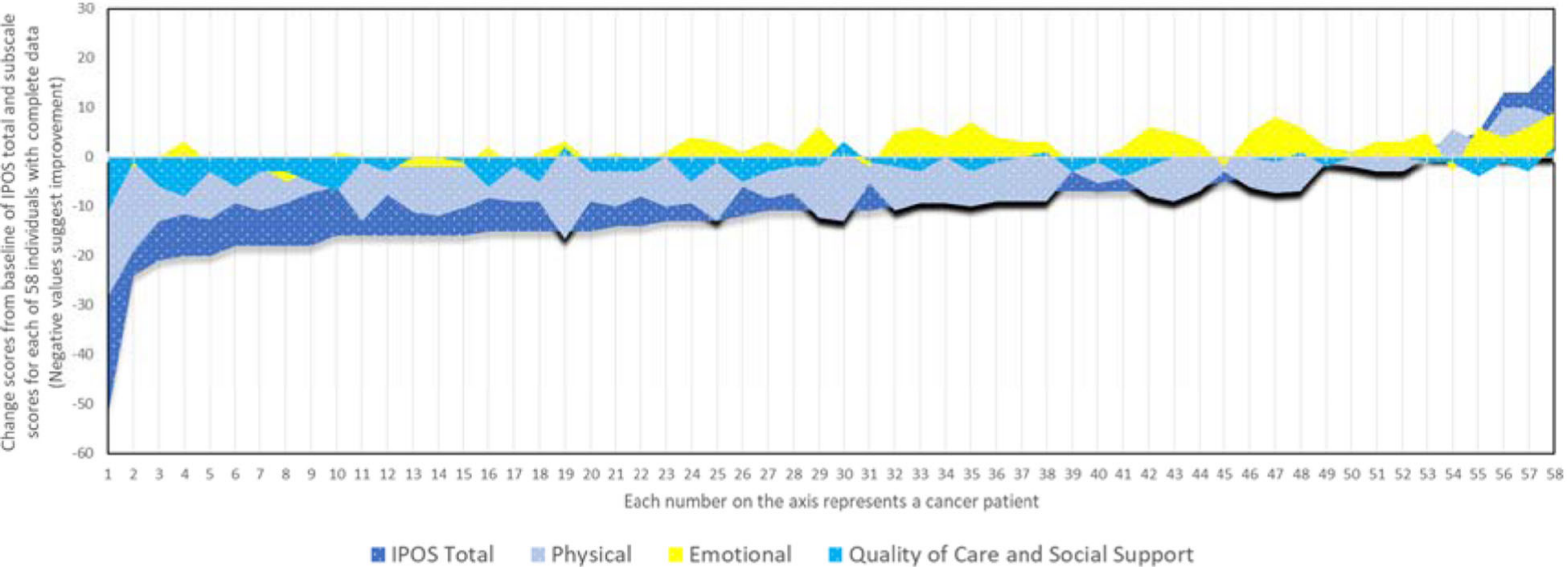
Internal responsiveness: Change scores from baseline for IPOS total and subscale scores (negative difference suggests improvement) (*n* = 58)

The mean time between the two administrations were 4.6 weeks (Range = 2–7, SD = 1.5). SRM of − 0.94 suggests large responsiveness [33]. Total scores at follow-up were significantly lower compared to baseline with a mean difference of 9.87 (7.18–12.58, 95% CI), t (57) = 7.33, *p* < 0.0001. A similar trend was observed for the physical (x_1_-x_2_ = 7.02, t (72) =10.3, *p* < 0.0001) and quality of care/social support subscales (x_1_-x_2_ = 2.11, t (61) =6.35, *p* < 0.0001). However, emotional concerns significantly worsened compared to baseline (x_1_-x_2_ = − 1.74, t (61) = − 4.2, *p* < 0.0001). Figure 3 maps change scores for 58 cancer patients with complete data across all four scales and assessments (Fig. 3).

## Discussion

In this study we adapted IPOS to Turkish and evaluated its cross-cultural validity and responsiveness in cancer patients. Most of the items were translated easily and perceived to be culturally relevant. Contrary to experiences of teams working on other language translations [16, 34], “at peace” item was particularly easy to adapt to Turkish as this is a very culturally relevant concept. Items on “practical problems addressed” and “information” were the most challenging to translate and adapt because the word “practical” is commonly used interchangeably to refer to “application”, and “information” can translate to “knowledge possessed”. Both items were clarified during cognitive testing.

Studies with cancer patients may be missing data [35] and higher rates of responses may be missing in palliative care studies compared to other studies [36]. Data for the baseline and follow-up assessment were impressively complete for most of the items. The baseline assessment of items on depression, information, anxiety of friends and family, and sharing feelings with friends and family generated the most non-responses. It is possible that patients may be struggling to come to terms with their cancer diagnosis or struggling to express the emotional impact of cancer on themselves, friends and family [37, 38]. Also, participants may have felt uncomfortable expressing their true responses to the item on “information” as not to offend the community palliative care staff caring for them, as was observed in the Japanese IPOS validation study [14].

Evaluation of SRMR and CFI values of the final CFA model could not confirm or negate that the three-domain structure proposed in the original IPOS was an acceptable fit to our data [39], in the original validation study 2-factor solution was implicated to be better fitting [11]. Even though CFI and SMRM parameters approached the minimums, they were not within the required defined parameters recommended for small samples. Future larger studies may be needed to confirm the three-domain structure. Emotional subscale, specifically had poorly loading items. Lack of consensus on cut off criteria for factor loadings in studies with small samples [40], necessities future larger studies before further actions are taken in relation to removal of items.

Turkish IPOS is acceptable, with no floor or ceiling effects. The mean and standard distribution of scores are consistent with findings from a recent validation study in cancer patients [14]. Turkish IPOS illustrated moderate to marginally moderate internal consistency and good to moderate reliability with its Physical and Social and Quality of Care subscales. However, the Emotional sub-scale showed poor internal consistency and reliability. These results agree with the findings in the original validation study [11], which reported low internal consistencies for Emotional and Social and Quality of Care subscales. In line with observations from the CFA, the removal of the item ‘Have any of your family or friends been anxious or worried about you?’ is indicated for improving internal consistency.

Content and face validity, ascertained through the translation and cross-cultural adaptation phase as well as at the validation phase, was also good as all of the main concerns such as feeling worried about treatment, the future, loss of independence and dying, the impact of the illness on the family, financial impact of illness, issues with getting support and pain were the main concerns brought up in the open-ended questions were covered in IPOS.

Construct validity was demonstrated with significant but moderate to low correlations with EQ-5D-3L items and VAS scale in the predicted direction. These findings were consistent with the findings of the Japanese IPOS [14]. Turkish IPOS also demonstrated known-groups validity as cancer patients at advanced stages had significantly higher total IPOS scores.

Turkish IPOS was able to detect statistically significant changes in concerns and symptoms between baseline and follow-up assessments. The effect size statistics were large. These findings supported internal responsiveness of the Turkish IPOS. The improvement in the total score was mostly due to significant improvement in the physical and quality of care/social support aspects. However, small but significant deterioration was reported with the emotional subscale. The general improvement or deterioration in the scores could be the effect of home palliative care services, but further studies are required to understand these changes in the context of clinical and subjective meaning [41].

There are further limitations of this study which need to be acknowledged. Patients were recruited through convenience sampling and may not be representative of all cancer patients. Additionally, patients receiving community palliative care services from the cancer charity may be more financially disadvantaged compared to the overall population of cancer patients. Patients with breast cancer were over-represented in the study as the charity was established by a breast cancer survivor and attracts more memberships among women living with breast cancer. Also, more women participated in the study as men may be less willing to share their experiences of having cancer [42]. These limitations may affect the generalizability of the findings.

The potential regional differences in Turkish language must also be taken into consideration. For this reason, minor amendments might be needed before Turkish IPOS is used in other Turkish speaking communities.

A team of researchers in Turkey have recently completed the cognitive testing of Turkish IPOS and have confirmed initiation of validation work with no revisions or modifications. Further research is needed to establish the range of Turkish IPOS scores that indicate clinical change meaningful to the patient and to establish its reliability and validity in non-cancer patient populations.

## Conclusion

Turkish IPOS is simple to complete and clearly understood. It measures the important aspects of needs and concerns that are relevant to Turkish speaking cancer patients and it is acceptable. Physical and Social and Quality of Care subscales are internally consistent and reliable, can be used to evaluate needs and concerns in these areas. There is good evidence that Turkish IPOS is a valid measure of concerns and needs, and responds to the severity of illness and is able to measure change over a specified time frame. Future studies are necessary to investigate the performance of the Emotional subscale, to clarify the factor structure and to evaluate the extent to which change in a IPOS relates to corresponding change in a reference measure of clinical or health status. Observing regional differences, Turkish IPOS may have implications for use in other Turkish speaking communities.

## Data Availability

The authors would like to declare that they have full control of all primary data and that we agree to allow the journal to review their data if requested.

## Abbreviations

CFA: Confirmatory Factor Analysis
CFI: Comparative Fit Index
COSMIN: Consensus-based Standards for the selection of health Measurement Instruments
IPOS: Integrated Palliative care Outcome Scale
KYHD: Help Those with Cancer Society in Cyprus
MECC: Middle Eastern Cancer Consortium
MVA: Missing Value Analysis
CPCT: Community Palliative Care Team
POS: Palliative care Outcome Scale
PROM: Patient-Reported Outcome Measure
SRMR: Standardized Root Mean Square Residual
VAS: Visual Analogue Scale

## References

[1] Bingley A, Clark D (2009). A comparative review of palliative care development in six countries represented by the Middle East cancer consortium (MECC). J Pain Symptom Manag.

[2] World Health Organization. WHO Definition of Palliative Care [Available from: https://www.who.int/cancer/palliative/definition/en/.

[3] Lynch T, Clark D, Centeno C, Rocafort J, de Lima L, Filbet M (2010). Barriers to the development of palliative care in Western Europe. Palliat Med.

[4] Costello J, Christoforou C (2001). Palliative care in a Mediterranean culture: a review of services in the Republic of Cyprus. Int J Palliat Nurs.

[5] European Commission (2017). State of health in the EU: Cyprus country health profile 2017.

[6] Temel JS, Greer JA, Muzikansky A, Gallagher ER, Admane S, Jackson VA (2010). Early palliative care for patients with metastatic non-small-cell lung cancer. N Engl J Med.

[7] Kıbrıs Postası. Sağlık Bakanlığı ile KHYD arasında palyatif bakım konusunda işbirliği protokolü imzalandı: Kibris Postasi; 2017. https://www.kibrispostasi.com/c77-SAGLIK/n234194-saglik-bakanligi-ile-khyd-arasinda-palyatif-bakim-konusunda-isbirligi-protokolu-imzalandi. Accessed Oct 2019.

[8] Gómez-Batiste X, Connor S (2017). Building integrated palliative care programs and services.

[9] Black N (2013). Patient reported outcome measures could help transform healthcare. Bmj.

[10] Bausewein C, Daveson BA, Currow DC, Downing J, Deliens L, Radbruch L (2016). EAPC white paper on outcome measurement in palliative care: improving practice, attaining outcomes and delivering quality services - recommendations from the European Association for Palliative Care (EAPC) task force on outcome measurement. Palliat Med.

[11] Murtagh FE, Ramsenthaler C, Firth A, Groeneveld EI, Lovell N, Simon ST (2019). A brief, patient- and proxy-reported outcome measure in advanced illness: validity, reliability and responsiveness of the integrated palliative care outcome scale (IPOS). Palliat Med.

[12] Sandham MH, Medvedev ON, Hedgecock E, Higginson IJ, Siegert RJ (2019). A Rasch analysis of the integrated palliative care outcome scale. J Pain Symptom Manag.

[13] Monnery D, Benson S, Griffiths A, Cadwallader C, Hampton-Matthews J, Coackley A (2018). Multi-professional-delivered enhanced supportive care improves quality of life for patients with incurable cancer. Int J Palliat Nurs.

[14] Sakurai H, Miyashita M, Imai K, Miyamoto S, Otani H, Oishi A (2019). Validation of the integrated palliative care outcome scale (IPOS) - Japanese version. Jpn J Clin Oncol.

[15] Veronese S, Rabitti E, Costantini M, Valle A, Higginson I (2019). Translation and cognitive testing of the Italian integrated palliative outcome scale (IPOS) among patients and healthcare professionals. PLoS One.

[16] Sterie AC, Bernard M (2019). Challenges in a six-phase process of questionnaire adaptation: findings from the French translation of the integrated palliative care outcome scale. BMC Palliat Care.

[17] Murtagh F, Ramsenthaler C, Firth A, Groeneveld EI, Lovell N, Simon S (2016). A brief, patient- and proxy-reported outcome measure for the adult palliative care population: validity and reliability of the integrated palliative outcome scale (IPOS). Palliat Med.

[18] OECD (2018). International migration outlook 2018.

[19] Schreiber JB (2008). Core reporting practices in structural equation modeling. Res Soc Adm Pharm.

[20] Mokkink LB, Terwee CB, Patrick DL, Alonso J, Stratford PW, Knol DL (2010). The COSMIN checklist for assessing the methodological quality of studies on measurement properties of health status measurement instruments: an international Delphi study. Qual Life Res.

[21] POS Development Team. [Available from: https://pos-pal.org.

[22] Polat U, Arpacı A, Demir S, Erdal S, Yalcin S (2014). Evaluation of quality of life and anxiety and depression levels in patients receiving chemotherapy for colorectal cancer: impact of patient education before treatment initiation. J Gastrointest Oncol.

[23] Antunes B, Daveson B, Ramsenthaler C, Benalia H, Lopes Ferreira P, Higginson I (2012). The palliative care outcome scale manual for cross-cultural adaptation and psychometric validation.

[24] Willis G, Leavy P (2015). Analysis of the cognitive interview in questionnaire design : understanding qualitative research.

[25] IBM SPSS (2017). Version 25.0.

[26] Albright JJ, Park HM. Confirmatory Factor Analysis Using Amos, LISREL, Mplus, and SAS/STAT CALIS. Working Paper. The University Information Technology Services (UITS) Center for Statistical and Mathematical Computing, Indiana University; 2009. http://www.indiana.edu/~statmath/stat/all/cfa/index.html.

[27] Kenny DA, McCoach DB (2003). Effect of the number of variables on measures of fit in structural equation modeling. Struct Equ Model Multidiscip J.

[28] Hu L, Bentler P (1999). Cutoff criteria for fit indexes in covariance structure analysis: conventional criteria versus new alternatives. Struct Equ Model.

[29] Cronbach LJ (1951). Coefficient alpha and the internal structure of tests. Psychometrika..

[30] Nunnally J, Bernstein I (1994). Psychometric theory.

[31] Koo TK, Li MY (2016). A guideline of selecting and reporting Intraclass correlation coefficients for reliability research. J Chiropr Med.

[32] Husted JA, Cook RJ, Farewell VT, Gladman DD (2000). Methods for assessing responsiveness: a critical review and recommendations. J Clin Epidemiol.

[33] Kazis LE, Anderson JJ, Meenan RF (1989). Effect sizes for interpreting changes in health status. Med Care.

[34] Beck I, Olsson Möller U, Malmström M, Klarare A, Samuelsson H, Lundh Hagelin C (2017). Translation and cultural adaptation of the integrated palliative care outcome scale including cognitive interviewing with patients and staff. BMC Palliat Care.

[35] Burton A, Altman DG (2004). Missing covariate data within cancer prognostic studies: a review of current reporting and proposed guidelines. Br J Cancer.

[36] Palmer JL (2004). Analysis of missing data in palliative care studies. J Pain Symptom Manag.

[37] de Graaff FM, Francke AL, van den Muijsenbergh ME, van der Geest S (2010). ‘Palliative care’: a contradiction in terms? A qualitative study of cancer patients with a Turkish or Moroccan background, their relatives and care providers. BMC Palliat Care.

[38] Goldstein D, Thewes B, Butow P (2002). Communicating in a multicultural society II: Greek community attitudes towards cancer in Australia. Intern Med J.

[39] Hooper D, Coughlan J, Mullen M (2008). Structural equation modelling: guidelines for determining model fit. Electron J Bus Res Methods.

[40] Shevlin M, Miles JNV (1998). Effects of sample size, model specification and factor loadings on the GFI in confirmatory factor analysis. Personal Individ Differ.

[41] Stratford PW, Binkley JM, Riddle DL (1996). Health status measures: strategies and analytic methods for assessing change scores. Phys Ther.

[42] Cecil R, Mc Caughan E, Parahoo K (2010). ‘It’s hard to take because I am a man’s man’: an ethnographic exploration of cancer and masculinity. Eur J Cancer Care.

